# A Topology-Based Metric for Measuring Term Similarity in the Gene Ontology

**DOI:** 10.1155/2012/975783

**Published:** 2012-05-15

**Authors:** Gaston K. Mazandu, Nicola J. Mulder

**Affiliations:** Computational Biology Group, Department of Clinical Laboratory Sciences, Institute of Infectious Disease and Molecular Medicine, University of Cape Town, Cape Town 7925, South Africa

## Abstract

The wide coverage and biological relevance of the Gene Ontology (GO), confirmed through its successful use in protein function prediction, have led to the growth in its popularity. In order to exploit the extent of biological knowledge that GO offers in describing genes or groups of genes, there is a need for an efficient, scalable similarity measure for GO terms and GO-annotated proteins. While several GO similarity measures exist, none adequately addresses all issues surrounding the design and usage of the ontology. We introduce a new metric for measuring the distance between two GO terms using the intrinsic topology of the GO-DAG, thus enabling the measurement of functional similarities between proteins based on their GO annotations. We assess the performance of this metric using a ROC analysis on human protein-protein interaction datasets and correlation coefficient analysis on the selected set of protein pairs from the CESSM online tool. This metric achieves good performance compared to the existing annotation-based GO measures. We used this new metric to assess functional similarity between orthologues, and show that it is effective at determining whether orthologues are annotated with similar functions and identifying cases where annotation is inconsistent between orthologues.

## 1. Introduction

 Worldwide DNA sequencing efforts have led to a rapid increase in sequence data in the public domain. Unfortunately, this has also yielded a lack of functional annotations for many newly sequenced genes and proteins. From 20% to 50% of genes within a genome [1] are still labeled unknown, uncharacterized, or hypothetical, and this limits our ability to exploit these data. Therefore, automatic genome annotation, which consists of assigning functions to genes and their products, has to be performed to ensure that maximal benefit is derived from these sequencing efforts. This requires a systematic description of the attributes of genes and proteins using a standardized syntax and semantics in a format that is human readable and understandable, as well as being interpretable computationally. The terms used for describing functional annotations should have definitions and be placed within a structure of relationships. Therefore, an ontology is required in order to represent annotations of known genes and proteins and to use these to predict functional annotations of those which are identified but as yet uncharacterized.

By capturing knowledge about a domain in a shareable and computationally accessible form, ontologies can provide defined and computable semantics about the domain knowledge they describe [2]. In biology, ontologies are expected to produce an efficient and standardized functional scheme for describing genes and gene products. Generally, such an ontology should be designed to cover a wide range of organisms, ensuring the integration of biological phenomena occurring in a wide variety of biological systems. In addition, it must be dynamic in nature in order to enable the design to incorporate new knowledge of gene and protein roles over time. One of the biggest accomplishments in this area is the creation of the gene ontology (GO) [3], which currently serves as the dominant and most popular functional classification scheme [4, 5] for functional representation and annotation of genes and their products. The construction of the gene ontology (GO) [3] arose from the necessity for organizing and unifying biology and information about genes and proteins shared by different organisms. At its outset, GO aims at producing a dynamic, structured and controlled vocabulary describing the role of genes and their products in any organism, thus allowing humans and computers to resolve language ambiguity.

GO provides three key biological aspects of genes and their products in a living cell, namely, complete description of the tasks that are carried out by individual proteins, their broad biological goals, and the subcellular components, or locations where the activities are taking place. GO consists of three distinct ontologies, molecular function (MF), biological process (BP), and cellular component (CC), each engineered as a directed acyclic graph (DAG), allowing a term (node) to have more than one parent. Traditionally, there were two types of relationships between a parent and a child. The “is_a" relation means that a child is a subclass or an instance of the parent, and the “part_of" relation indicates the child is a component of a parent. Thus, each edge in a GO-DAG represents either an “is_a” or a “part_of” association. However, another relationship has emerged, namely, “regulates”, which includes “positively_regulates” and “negatively_regulates”, and provides for relationships between regulatory terms and their regulated parents [6]. As we are only interested in the GO-DAG topology in the sense that where a term occurs, its parents also occur, regardless of whether the term regulates the parent term or not, we only use the relations “is_a” and “part_of” here, and these are treated equally. The is_a relationships are more prominent, constituting approximately 88% for BP, 99% for MF, and 81% for CC, of all the relationships, so the impacts of part_of relationships are less significant.

The GO has been widely used and deployed in several protein function prediction analyses in genomics and proteomics. This growth in popularity is mainly due to the fundamental organization principles and functional aspects of its conception displayed by its wide coverage and biological relevance. Specific tools, such as the AmiGO browser [7, 8], have been developed for making GO easy to use and have significantly contributed to the large expansion of GO in the experimental and computational biology fields. Nowadays, GO is the most widely adopted ontology by the life science community [9], and this superiority has been proven by successes resulting from its use in protein function prediction. The GO annotation (GOA-UniProtKB) project arose in order to provide high-quality annotations to gene products and is applied in the UniProt knowledgebase (UniProtKB) [10–13]. It also provides a central dataset for annotation in other major multispecies databases, such as Ensembl and NCBI [14].

Considering its wide use, the issues related to its design and usage have been qualified as critical points [15] to be taken into account for effectively deploying GO in genome annotation or analysis. One of the issues is associated with the depth of GO, which often reflects the vagaries in different levels of biological knowledge, rather than anything intrinsic about the terms [2]. Consequently, two genes or proteins may be functionally similar but technically annotated with different GO Ids. Although several approaches have been designed to assess the similarity and correlation between genes [16–21] using their sequences or gene expression patterns from high-throughput biology technologies, some methods exist for measuring functional similarities of genes based on their GO annotations but these have their drawbacks. An effective approach should be able to consider the issue related to the depth of the GO-DAG raised previously and provide a clear relation of how similar a parent and child are using only the GO-DAG topology. This should apply to gene or protein GO annotations derived from different sources and be independent of the size of the GO-DAG, as GO is still expanding.

Several GO term similarity measures have been proposed for characterizing similar terms, each having its own strengths and weaknesses. These similarity measures are partitioned into edge- and node-based approaches according to Pesquita et al. [9]. Edge-based similarity measures are based mainly on counting the number of edges in the graph to get the path between two terms [22, 23]. Among them, we have the longest shared path (LP) approach implemented in the GOstats package of Bioconductor [24] and the IntelliGO approach suggested by Benabderrahmane et al. [25]. Although these approaches use only the intrinsic structure of the hierarchy under consideration, they generally suffer from the fact that they consider only the distance between terms, ignoring their position characteristics within the hierarchy. Thus, nodes at the same level have the same semantic distance to the root of the hierarchy, producing a biased semantic similarity between terms. In order to alleviate this issue, edges can be weighted differently depending on their level in the hierarchy to influence the similarity scores [26]. Unfortunately, using these edge weighting approaches does not completely resolve the problem [9]. The node-based approaches use the concept of information content, also called semantic value, to compare the properties of the terms themselves and relations to their ancestors or descendants, and these measures are referred to as IC-based (information content-based) approaches [27].

Here we introduce a new semantic similarity measure of GO terms based only on the GO-DAG topology to determine functional closeness of genes and their products based on the semantic similarity of GO terms used to annotate them. This measure incorporates position characteristic parameters of GO terms to provide an unequivocal difference between more general terms at the higher level, or closer to the root, and more specific terms at the lower level, or further from root node. This provides a clearer topological relationship between terms in the hierarchical structure. This new measure is a hybrid node- and edge-based approach, overcoming not only the issue related to the GO-DAG depth, as stated previously, but also the issues related to the dependence on the annotation statistics of node-based approaches and those related to edge-based approaches in which nodes and edges at the same level are evenly distributed.

## 2. Materials and Methods

 In this section we survey existing annotation- and topology-based approaches and set up a novel GO semantic similarity metric in order to measure GO term closeness in the hierarchy of the GO-directed acyclic graph (DAG). This novel GO term semantic similarity measure is derived in order to ensure effective exploitation of the large amounts of biological knowledge that GO offers. This, in turn, provides a measurement of functional similarity of proteins on the basis of their annotations from heterogeneous data using semantic similarities of their GO terms.

### 2.1. Existing GO-IC-Based Semantic Similarity Approaches

 We are interested in the IC-based approaches, and unlike the graph-based or hybrid approach introduced by Wang et al. [28], which is based on the intrinsic structure of the GO-DAG, that is, only uses the GO-DAG topology to compute the semantic similarity, other measures do not consider only the topology. Most of them are adapted from Resnik [29] or Lin's [30] methods, in which the information content (or semantic value) of a term conveying its biological description and specificity is based on the annotation statistics related to the term [2, 31], and thus they have a natural singularity problem caused by orphan terms. Here these approaches are referred to as Resnik-related approaches. In these approaches, the more often the term is used for annotation, the lower its semantic value, and as pointed out by Wang et al., this may lead to different semantic values of the GO terms for GO annotation data derived from different sources. However, each biological term in the ontology is expected to have a fixed semantic value when used in genome annotation. The semantic value is defined as the biological content of a given term, and this is particularly a problem in the hierarchical structure of the GO-DAG if the information will be used to predict functions of uncharacterized proteins in the genome, since one source can annotate a given protein with a term at a low level and another source with a term at a higher level in the hierarchy. Furthermore, the description and specificity of a given term in GO essentially depends on its GO annotation specification, translated by its position in the GO-DAG structure or topology.

To overcome these limitations, Wang introduced a topology-based semantic similarity measure in which the semantic value of a term *z* is given by

(1) $$\text{I}{\text{C}}_{W}\left(z\right)=\underset{t\in T_{z}}{\sum }S_{z}\left(t\right),$$

where *T*_*z*_ denotes the set of ancestors of the term *z* including *z*, and *S*_*z*_(*t*) is calculated as follows:

(2) $$S_{z}\left(t\right)=\{\begin{array}{cc} 1, & \text{if}\;t=z,\;\\ \max\left\{\omega_{e}*S_{z}\left(t^{\prime }\right):t^{\prime }\in {𝒞}_{h}\left(t\right)\right\}, & \text{otherwise},\; \end{array}$$

with *𝒞*_*h*_(*t*) being the set of children of the term *t*, and *ω*_*e*_ the semantic contribution factor for “is_a” and “part_a” relations set to 0.8 and 0.6, respectively. The semantic similarity of the two GO terms is given by

(3) $$S_{W}\left(x,y\right)=\frac{\sum_{t\in T_{x}\cap T_{y}}\left(S_{x}\left(t\right)+S_{y}\left(t\right)\right)}{\text{I}{\text{C}}_{W}\left(x\right)+\text{I}{\text{C}}_{W}\left(y\right)}.$$

It has been shown that the Wang et al. approach performs better than Resnik's approach in clustering gene pairs according to their semantic similarity [27, 28].

On the edge-based similarity approaches, Zhang et al. [32] introduced a GO-topology-based approach to assess protein functional similarity for retrieving functionally related proteins from a specific proteome, overcoming the common issue of other edge-based approaches mentioned previously. This was achieved by computing a measure called the *D* value, which depends only on the children of a given GO term and is numerically equal to the sum of *D* values of all its children. Thus, the *D* value of a GO term is calculated using a recursive formula starting from leaves in the hierarchical structure, where the *D*-value of all leaves are equal and set to the inverse multiplicative of the count of the root obtained by recursively summing the counts of all the direct children from the bottom up, with the count of the leaf set to 1. Note that the count of a given nonleaf term is just the number of all paths from that term node to all leaves connected to the term. In this approach, the *D* value for a pair of terms *x* and *y* is given by

(4) D(x,y)=min⁡{D(z):z∈𝒜(x,y)}.

However, a general limitation common to all these semantic similarity measures is that none of them fully address the issue related to the depth of the GO-DAG as stated previously; that is, the depth sometimes reflects vagaries in different levels of knowledge. An example is where the structure is just growing deeper in one path without spreading sideways. In the context of the GO-DAG, such a term is sometimes declared obsolete and automatically replaced by its parent. Thus, to consider this issue, we are introducing a topological identity or synonym term measure based on term topological information in which a parent term having only one child and that child term having only that parent are assumed to be topologically identical and they are assigned the same semantic value. This provides an absolute difference between more general terms closer to the root and more specific terms further from the root node, depending on the topology of the GO-DAG, that is, whether a branch splits into more than one possible path of specificity. Furthermore, this is consistent with the human language in which the semantic similarity between a parent term and its child depends on the number of children that the parent term possesses and also the number of parents that the child term has. Intuitively a parent having more children loses specificity and this parent is no longer relevant to be used for its child specification, thus leading to a lower similarity score between this parent and each of its children.

**Figure 1:**
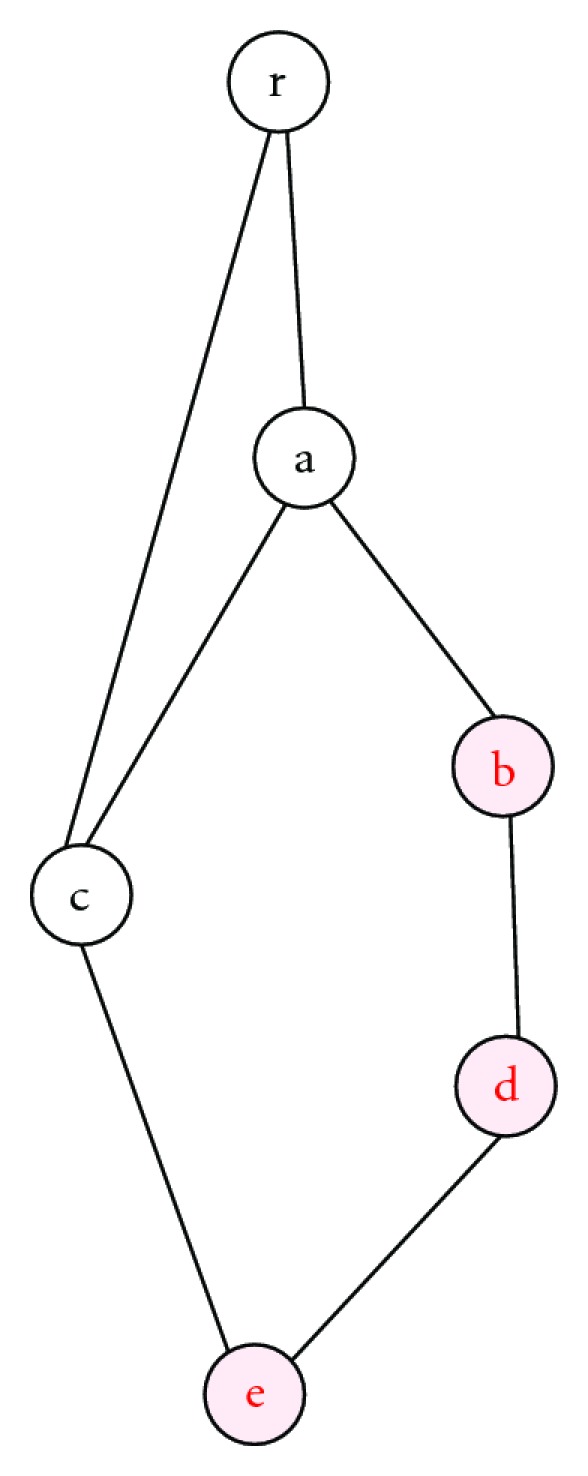
Fictitious hierarchical structure illustrating the computation of term semantic values. Terms are nodes with “r” as a root.

To illustrate this, let us consider the hierarchical structure in Figure 1 where “a”, “b”, “c”, “d”, and “e” are terms used to annotate proteins in a given genome and these terms are linked by the relation “is_a”. For the Zhang et al. approach, the semantic values of “b” and “d” are the same, which is 1.09861 (−ln⁡(1/3)), but it fails to distinguish between “d” and “e”, which would be expected to have different semantic values. The Wang et al. approach will assign different semantic values to “b” and “d”; the semantic value of “b” is 2.44 and that of “d” is 2.952, although they are topologically identical in the sense that there is no other option going down the DAG except to “d”. For annotation-based approaches, if we consider a genome, for example, which has been annotated by two different labs, referred to as heterogeneous sources, it is likely that the terms “b” and “d” will not occur at the same frequency, in which case “b” and “d” will have different semantic values. For this new measure, the term “b” has only one child “d”, which has only one parent “b” (no sideways spread) and therefore the term “d” does not have additional value compared to “b” in the illustration in Figure 1. This means that “b” and “d” are topologically identical (synonymous) and have the same fixed semantic value, equal to 1.38629. This is different to the semantic value of the term “e”, which is 3.46574 as “e” could be “derived” from two different branches.

### 2.2. GO Term Topological Information and New GO Term Similarity Approach

 Translating the biological content of a given GO term into a numeric value, called the semantic value or topological information, on the basis of its location in the GO-DAG, requires knowledge of the topological position characteristics of its immediate parents. This leads to a recursive formula for measuring topological information of a given GO term, in which the child is expected to be more specific than its parents. The more children a term has, the more specific its children are compared to that term, and the greater the biological difference. In addition, the more parents a term has, the greater the biological difference between this term and each of its parent terms. The three separate ontologies, namely, molecular function (MF), biological process (BP), and cellular component (CC) with GO Ids GO: 0003674, GO: 0008150, and GO: 0005575 respectively, are roots for the complete ontology, located at level 0, the reference level, and are assumed to be biologically meaningless. Unless specified explicitly, in the rest of this work the level of a term is considered to be the length of the longest path from the root down to that term in order to avoid a given term and its child having the same level. *𝒩*_GO_ and *ℒ*_GO_ will, respectively, express the set of GO terms and links, (*x*, *y*) ∈ *ℒ*_GO_ represents the link or association between a given parent *x* and its child *y*, and the level of the link (*x*, *y*) is the level of its source node *x*. Finally, [*x*, *y*] ∈ *𝒩*_GO_ indicates that the level of term *x* is lower than that of *y*.

**Definition 1**

The topological information IC_*T*_(*z*) of a given term *z* ∈ *𝒩*_GO_ is computed as

(5) $$\text{I}{\text{C}}_{T}\left(z\right)=-\ln\left(\mu \left(z\right)\right),$$

where *μ*(*z*) is a topological position characteristic of *z*, recursively obtained using its parents gathered in the set *𝒫*_*z*_ = {*x* : (*x*, *z*) ∈ *ℒ*_GO_}, and given by

(6) $$\mu \left(z\right)=\{\begin{array}{cc} 1, & \text{if}\;z\;\text{is a root,}\;\\ \underset{x\in {𝒫}_{z}}{\prod }\frac{\mu \left(x\right)}{{𝒞}_{x}}, & \text{otherwise,}\; \end{array}$$

with *𝒞*_*x*_ being the number of children of parent term *x*.

A topological position is thus a function *μ* : *𝒩*_GO_ → [0,1], such that for any term *t* ∈ *𝒩*_GO_, *μ*(*t*) defines a reachability measure of an instance of term *t*. Obviously, *μ* is monotonically increasing as one moves towards the root; that is, if *t*_1_ is_a *t*_2_, then *μ*(*t*_1_) ≤ *μ*(*t*_2_). For the top node or root, the reachability measure is 1. Furthermore, this reachability measure takes into account information of parents of the term under consideration through their reachability measures and that of every parent's children by incorporating the number of children that each parent term has in order to quantify how specific a given child is compared to each of its parent terms.

Note that, in general, the information we possess about something is a measure of how well we understand it and how well ordered it is. *μ*(*z*) provides a precise indicator of all we know about the term *z* in the DAG structure. As *μ* is decreasing when moving towards leaves and a strictly positive defined function, the multiplicative inverse of *μ* is an increasing function. This implies that 1/*μ*(*z*) is a measure of how we understand the term *z* and how ordered it is in the DAG, which merely means that the inverse of *μ*(*z*) measures the information we possess about the term *z* in the context of the DAG structure. The formula in (5) is a logarithmic weighting of the inverse of *μ*(*z*), referred to as topological information and measuring what we know about the term *z* in the DAG structure.

**Figure 2:**
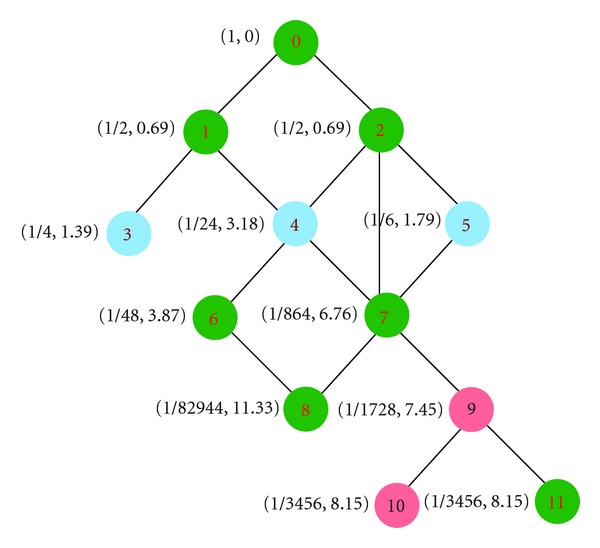
Hierarchical structure illustrating how our approach works. Nodes are represented by integers from 0 to 11 with 0 as a root. The numbers beside each node represent its topological position characteristic and information content.

To illustrate the way this approach works, consider the hierarchical structure shown in Figure 2. In this DAG from top to bottom, we have the following.

- The topological position characteristic of the root 0 is *μ*(0) = 1, and so its topological information is IC_*T*_(1) = −ln⁡(1) = 0.
- As 1 and 2 have only parent 0, which has only these two children with *μ*(0) = 1, this yields *μ*(1) = 1/2 = *μ*(2), and so their topological information is IC_*T*_(1) = −ln⁡(1/2) = 0.69315 = IC_*T*_(2).
- 3 has only one direct parent 1 with *μ*(1) = 1/2 and this parent has two children, we have *μ*(3) = 1/4, and its topological information is then IC_*T*_(3) = −ln⁡(1/4) = 1.38639.
- 4 has two direct parents 1 and 2. 1 has two children with *μ*(1) = 1/2 and 2 has three children with *μ*(2) = 1/2. Thus, its topological position characteristic is the product of topological position characteristics of its parents, respectively, divided by the number of children for each parent *μ*(4) = 1/4∗1/6 = 1/24 and its topological information is IC_*T*_(4) = −ln⁡(1/24) = 3.17806.
- 5 has only one direct parent 2, which has three children and *μ*(2) = 1/2. Its topological position characteristic is *μ*(5) = 1/6 and its topological information is IC_*T*_ = −ln⁡(1/6) = 1.79176.

Unlike edge-based approaches where nodes and edges are uniformly distributed, and edges at the same level of the ontology correspond to the same semantic distance between terms [9], in this new approach these parameters depend on the topological position characteristic of terms, which are not necessarily the same. In this illustration, nodes 3, 4, and 5 are at the same level but they do not have the same topological position characteristic, thus leading to different topological information or semantic values. Furthermore, the aforemetioned illustration reveals that the product in formula (6) of topological position characteristic must be carefully considered when implementing the approach, since the exponential tail-off with increasing depth is severe depending on the density of the hierarchical structure under consideration. Here, we suggest computing *μ*(*z*) iteratively when performing this product, and every time the multiplication is done, the obtained value must immediately be converted to a pair of numbers (*α*, *β*) such that *μ*(*z*) = *α*10^*β*^ with 0.1 ≤ *α* < 1 and *β* < 0. This means that every time the product is performed, the new value is converted to this format so that in the end, the topological position characteristic is just given by (*α*, *β*) such that *μ*(*z*) = *α*10^*β*^ and IC_*T*_ = −ln⁡(*α*) − *β*ln⁡(10).

**Definition 2**

Let [*x*, *y*] ∈ *𝒩*_GO_; *x* and *y* are topologically identical or synonym terms and denoted by $x\overset{\text{GO}}{=}y$, if the following properties are satisfied.

- IC_*T*_(*x*) = IC_*T*_(*y*) or *μ*(*x*) = *μ*(*y*).
- There exists one path *p*_*xy*_ from *x* to *y*.

Therefore, two GO terms are equal if and only if they are either the same or topologically identical terms. Suppose that there exists a path *p*_*xy*_ from term *x* to term *y*, *x* is a more general term compared to *y*, or *y* is more specific compared to *x* and denoted by $x\overset{\text{GO}}{<}y$ if IC_*T*_(*x*) < *I*C_*T*_(*y*) or *μ*(*y*) < *μ*(*x*).

The topological position *μ* provides a new way of assessing the intrinsic closeness of GO terms. Two terms in the GO-DAG may share multiple ancestors as a GO term can have several parents through multiple paths. Therefore, we define the topological position *μ*_*s*_(*x*, *y*) of *x* and *y* as that of their common ancestor with the smallest topological position characteristic, that is,

(7) $$\mu_{s}\left(x,y\right)=\min\left\{\mu \left(t\right):t\in 𝒜\left(x,y\right)\right\},$$

where *𝒜*(*x*, *y*) = *𝒜* ∪ {*x*, *y*} with *𝒜* being the set of ancestral terms shared by both terms *x* and *y*. Finally, the semantic similarity score of the two GO terms is given by

(8) $$S_{\text{GO}}\left(x,y\right)=\frac{\text{I}{\text{C}}_{T}\left(x,y\right)}{\max\left\{\text{I}{\text{C}}_{T}\left(x\right),\text{I}{\text{C}}_{T}\left(y\right)\right\}},$$

with IC_*T*_(*x*, *y*) = −ln⁡*μ*_*s*_(*x*, *y*) being the topological information shared by the two concepts *x* and *y*.

The semantic similarity measure *S*_GO_ proposed here is referred to as the GO-universal similarity measure [33], as it induces a distance or a metric, *d*_GO_, given by *d*_GO_(*x*, *y*) = 1 − *S*_GO_(*x*, *y*) (see Supplementary Material available online at doi:10.1155/2012/975783), which in Information Theory is known as a universal metric [34]. The more topological information two concepts share, the smaller their distance and the more similar they are. Moreover, the similarity formula in (8) emphasizes the importance of the shared GO terms by giving more weight to the shared ancestors corrected by the maximum topological information, and thus measuring how similar each GO term is to the other. Thus, for two GO terms sharing less informative ancestors the distance is greater and the similarity is smaller, while for two GO terms sharing more informative ancestors, they are closer and their similarity is higher.

**Figure 3:**
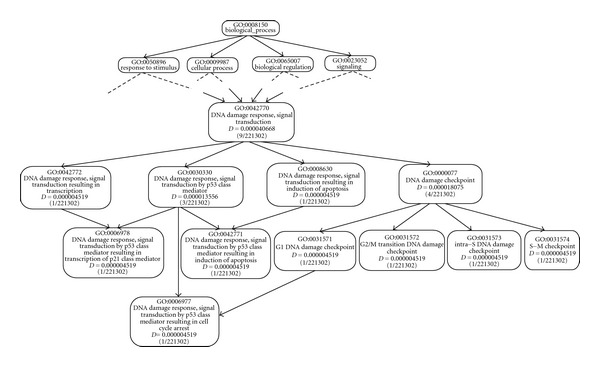
Subgraph of the GO BP. Each box represents a GO term with GO ID, *D* value (Zhang et al. measure). This is used to illustrate our approach and compare its effectiveness to the Zhang et al. approach.

To illustrate the GO-universal approach, we use (5) and (6) to compute the reachability measure *μ*(*z*) and topological information measure IC_*T*_(*z*) of GO terms *z* in a minimum spanning graph shown in Figure 3 adapted from [32]. Results are shown in Table 1 for our approach and the Zhang et al. approach. To relate the scale of Zhang et al. to ours, the *D* value of a given term is considered to be the probability of usage or occurrence of the term in the structure as suggested by Zhang et al. This means that the information content (IC) of a term *x* is calculated as

(9) $$\text{I}{\text{C}}_{Z}\left(x\right)=-\ln\left(D\left(x\right)\right).$$

Moreover, two approaches, Resnik and Lin's approaches, are used for scaling the semantic similarity measure induced by IC_*Z*_ between 0 and 1. The uniform Resnik's measure is given by

(10) $$S_{ZuR}\left(x,y\right)=\max\left\{\text{I}{\text{C}}_{Zu}\left(a\right):a\in 𝒜\left(x,y\right)\right\},$$

where IC_*Zu*_(*a*) is the uniform IC_*Z*_(*a*) obtained by dividing IC_*Z*_(*a*) by the maximum scale whose value is ln⁡*N* where *N* is the total number of terms within the ontology under consideration. IC_*Zu*_(*a*) is therefore computed as follows:

(11) $$\text{I}{\text{C}}_{Zu}\left(a\right)=\frac{\text{I}{\text{C}}_{Z}\left(a\right)}{\ln\;N},$$

where *N* is the number of terms in the ontology under consideration. Lin's semantic similarity measure is given by

(12) $$S_{ZL}\left(x,y\right)=\max\left\{\frac{2\times \text{I}{\text{C}}_{Z}\left(a\right)}{\text{I}{\text{C}}_{Z}\left(x\right)+\text{I}{\text{C}}_{Z}\left(y\right)}:a\in 𝒜\left(x,y\right)\right\}.$$

As we can see, the more specific the term, that is, the further it is from the root node, the higher its topological information, meaning that children are more informative or more specific than their parents, and for two GO terms in the same path, the more specific one will either be more informative or topologically identical to that closer to the root. This is not the case for the Zhang et al. approach, in which the semantic values of the terms at the same level tend to be uniform and a child term is not necessarily more specific than a given parent term, independent of the number of parents that the child term has. Our method distinguishes these different local topologies.

We calculate the semantic similarity between every two consecutive GO terms in Figure 3 and results are given in Table 2 for three different approaches. The formula in (6) shows that, for our approach, the contribution of a given parent to the term depends on the parent reachability measure. The smaller the reachability measure of that parent and the fewer children it possesses, the higher its similarity compared to another parent of the term. From the results in Table 2, we see that GO:0042771 is more similar to GO:0008630 than to GO:0030330, both of which are its parents. This is topologically explained by the lower reachability of GO:0008630 compared to GO:0030330 and the higher number of children the term GO:0030330 possesses. This reduces its influence on each of its children, becoming less relevant for it to represent a given child due to the lower similarity between them. Furthermore, GO:0006977 is more similar to GO:0031571 than to GO:0030330. This is numerically due to the influence of GO:0030330, reflected by its reachability measure, which is lower than that of GO:0031571. It is topologically caused by the higher level of the term GO:0031571 compared to the level of GO:0030330, and therefore gives the term GO:0031571 a higher biological content property than GO:0030330 for better representing the child term GO:0006977.

Table 2 also includes the semantic similarity between every two consecutive GO terms computed using the Zhang et al. and Wang et al. methods. These results show that Wang's semantic similarity measure between a given term and its immediate child is always greater than 0.6, which is the semantic factor of “part_of” relations, and is independent of the characteristics of the position of these terms in the GO-DAG, including the number of children belonging to the parent term and their levels. This shows how our approach provides a scalable and consistent measurement method, in which the semantic similarity of two terms is completely determined by their reachability measures and that of their highest informative ancestor, that is, the ancestor with the smallest reachability measure. Using the intrinsic topology property of the GO-DAG, the semantic similarity measure of two terms is in agreement with the GO consortium vocabulary, in the sense that two terms whose most common informative ancestor is close to the root share less topological information compared to those having the highest common informative ancestor far from the root.

### 2.3. Functional Similarity of Proteins Based on GO Similarity

 A given protein may perform several functions, thus requiring several GO terms to describe these functions. For characterized or annotated pairwise proteins with known GO terms, functional closeness or GO similarities based on their annotations and consequently the distances between these proteins can be evaluated using the Czekanowski-Dice approach [35] as follows:

(13) $$\begin{array}{cc} & S_{ℱ}\left(p_{1},p_{2}\right)\\ & \;\;=\frac{2\times \left|T_{\text{GO}}^{X}\left(p_{1}\right)\cap T_{\text{GO}}^{X}\left(p_{2}\right)\right|}{\left|T_{\text{GO}}^{X}\left(p_{1}\right)\cup T_{\text{GO}}^{X}\left(p_{2}\right)\right|+\left|T_{\text{GO}}^{X}\left(p_{1}\right)\cap T_{\text{GO}}^{X}\left(p_{2}\right)\right|}, \end{array}$$

where *T*_GO_^*X*^(*p*) is the set of GO terms of a given protein *p* for a given ontology *X* = MF, BP, CC, and |*T*_GO_^*X*^(*p*)| stands for its number of elements.

Czekanowski-Dice's measure is not convenient for using in the case of GO term sets, since GO terms may be similar at some level without being identical. This aspect cannot be captured in Czekanowski-Dice's measure which only requires the contribution from the GO terms exactly matched between the sets of GO terms of these proteins. One can attempt to avoid this difficulty by incorporating the true path rule in the computation of the intersection and union of GO term sets for proteins. However, in most cases where these proteins are annotated by successive GO terms in the GO-DAG, this may lead to the situation where the number of elements in the union of these sets is equal to that of their intersection plus one, in which case, the functional closeness of these proteins is forced to converge to 1, independently of the biological contents of the GO terms in the GO-DAG.

To overcome this problem, we set up a functional similarity between proteins which emphasizes semantic similarity between terms in their sets of GO terms considered to be uniformly distributed. This functional similarity is given by

(14) $$\begin{array}{cc} S_{ℱ}\left(p_{1},p_{2}\right) & =\frac{1}{2}[\frac{1}{\left|T_{\text{GO}}^{X}\left(p_{1}\right)\right|}\underset{t\in T_{\text{GO}}^{X}\left(p_{1}\right)}{\sum }S_{\text{GO}}\left(t,T_{\text{GO}}^{X}\left(p_{2}\right)\right)\\ & \;\;\;+\frac{1}{\left|T_{\text{GO}}^{X}\left(p_{2}\right)\right|}\underset{t\in T_{\text{GO}}^{X}\left(p_{2}\right)}{\sum }S_{\text{GO}}\left(t,T_{\text{GO}}^{X}\left(p_{1}\right)\right)], \end{array}$$

where *S*_GO_(*t*, *T*_GO_^*X*^(*p*)) = 1 − *d*_GO_(*t*, *T*_GO_^*X*^(*p*)), with *d*_GO_(*t*, *T*_GO_^*X*^(*p*)) being the distance between a given term *t* and a set of terms *T*_GO_^*X*^(*p*) for a given protein *p*, mathematically defined as follows:

(15) $$d_{\text{GO}}\left(t,T_{\text{GO}}^{X}\left(p\right)\right)=\min\left\{d_{\text{GO}}\left(t,s\right):s\in T_{\text{GO}}^{X}\left(p\right)\right\}.$$

Thus, owing to the fact that *d*_GO_(*s*, *t*) = 1 − *S*_GO_(*t*, *s*), we obtain

(16) $$S_{\text{GO}}\left(t,T_{\text{GO}}^{X}\left(p\right)\right)=\max\left\{S_{\text{GO}}\left(t,s\right):s\in T_{\text{GO}}^{X}\left(p\right)\right\}.$$

This shows that the functional closeness formula emphasizes the importance of the shared GO terms by assigning more weight to similarities than differences. Thus, for two proteins that do not share any similar GO terms, the functional closeness value is 0, while for two proteins sharing exactly the same set of GO terms, the functional closeness value is 1. The functional similarity between proteins in (14) is a value that ranges between 0 and 1 and indicates the percentage of similarity the two proteins share, on average, based on their annotations. For example, a functional similarity between two proteins of 0.9 means that these proteins are 90% similar, on average, based on their annotations.

Note that the approach used here to combine GO term topological information for calculating protein functional similarity scores was used in the context of annotation-based approaches and is referred to as the best match average (BMA) approach. This approach has been suggested to be better than the average (Avg) [2] or maximum (Max) [19] approaches from a biological point of view [36, 37]. However, even Avg and Max approaches can also be used to combine GO term semantic similarity scores produced using this new measure to quantify protein functional similarity depending on the application. Furthermore, the GO-universal metric can be used in the context of the SimGIC approach [9, 38] derived from the Jaccard index based on the Tversky ratio model of similarity [39], which uses GO term IC directly in order to compute protein functional similarity scores, and referred to as SimUIC. These approaches are generally referred to as term-based approaches. The GO term topological information scores can also be used to construct protein functional similarity schemes relying on other Tversky ratio models, for example, using the Dice index, referred to as SimDIC, and SimUIX which uses a universal index, given by

(17) $$\begin{array}{cc} \text{SimDIC}\left(p,q\right) & =\frac{2\times \sum_{x\in T_{\text{GO}}^{X}(p)\cap T_{\text{GO}}^{X}(q)}\text{I}{\text{C}}_{T}\left(x\right)}{\sum_{x\in T_{\text{GO}}^{X}(p)}\text{I}{\text{C}}_{T}\left(x\right)+\sum_{x\in T_{\text{GO}}^{X}(q)}\text{I}{\text{C}}_{T}\left(x\right)},\\ \text{SimUIX}\left(p,q\right) & =\frac{\sum_{x\in T_{\text{GO}}^{X}(p)\cap T_{\text{GO}}^{X}(q)}\text{I}{\text{C}}_{T}\left(x\right)}{\max\left\{\sum_{x\in T_{\text{GO}}^{X}(p)}\text{I}{\text{C}}_{T}\left(x\right),\sum_{x\in T_{\text{GO}}^{X}(q)}\text{I}{\text{C}}_{T}\left(x\right)\right\}}. \end{array}$$

## 3. Results and Discussion

 We have developed a semantic value measurement approach for GO terms using the intrinsic topology of the GO-DAG and taking into account issues related to the depth of the structure. We evaluate our method against the Wang et al. and Zhang et al. topology-based methods for a specific subgraph of the GO-DAG and then use UniProt data to compare our similarity scores to those of annotation-based approaches. Note that the Zhang et al. approach has recently been shown to perform equally to the Resnik measure and to perform better than the Wang et al. measure [40] and the relevance approach which is the Lin enhancement measure suggested by Schlicker et al. [31].

### 3.1. Evaluation of the New Approach

 We have seen Section 2 that the GO-universal similarity measure produces effective semantic similarity scores based on the intrinsic topology of the GO-DAG by making explicit use of topological relationships between different terms, thus producing a clearer representation of these relations. As discussed previously, the biggest limitation of existing approaches based on Resnik's algorithm is that they are constrained by the annotation statistics related to the terms. On the other hand, although, like ours, Wang's measure is based only on the intrinsic topology of the GO-DAG, one of the drawbacks of their approach is that it raises a scalability issue since it requires complete knowledge of the sub-GO-DAG of the two terms for which the semantic similarity is being computed and that of all their common ancestors. However, since GO is expanding and increasing in size, the term relationships are becoming more and more important. Thus, a semantic similarity measurement approach should be effective independent of the size of the GO-DAG.

Another negative aspect of Wang's approach is that it essentially relies on the semantic factors of “is_a” and “part_of” relations, and it is not clear for which values of these semantic factors the semantic similarity measure yields the optimal value of biological content of terms. Moreover, these semantic factors make the similarity value between a given child and its direct parent independent of the number of children that the parent term has (shown in (3)). Wang's semantic similarity measure between a given term and its immediate child term depends solely on the semantic relationship (“part_of” or “is_a”) and is completely independent of the position characteristics in the hierarchical structure. However, considering the GO-DAG, the semantic similarity between a given term and its child should not only depend on the number of parents the child term possesses, but also on the number of children that the parent term possesses. The more children a term has, the smaller the semantic similarity to each of its children, which is logical.

The Zhang approach, which depends only on the children of a given term, often fails to effectively differentiate a child from its parents, yielding an equal *D* value and IC for these terms. It also tends to produce a uniform semantic similarity between a parent and its children (see Table 2 in Section 2), which is overestimated to 1 when using Lin's approach, whereas these GO terms are biologically and topologically different. This means that the approach ignores the fact that a child is more specific than the parent by assigning them the same semantic value and consequently the approach fails to distinguish proteins annotated by these terms, which leads to an overestimation of functional similarity between these proteins. This case occurs, for instance, for the child-parent GO terms: GO:0006978 and GO:0042772, GO:0042771 and GO:0008630, and GO:0006977 and GO:0031571, all of which have identical values. These observations suggest that a given similarity approach relying on the intrinsic topology of the hierarchical structure should consider both GO term parents and children in its conception.

### 3.2. Performance Evaluation of the GO-Universal Metric

 We first evaluated the performance of the new metric by assessing its ability to capture functional coherence in a human protein-protein interaction network in terms of how interacting proteins are functionally related to each other. Expert-curated and experimentally determined human protein-protein interactions (PPIs) were retrieved from the IntAct database [41], the Database of Interacting Proteins (DIP) [42], the Biomolecular Interaction Network Database (BIND) [43], the Mammalian Protein-Protein Interaction Database (MIPS) [44], the Molecular INTeraction database (MINT) [45], and the Biological General Repository for Interaction Datasets (BioGRIDs) [46]. These networks were integrated into a single network where we only considered interactions predicted by at least two different approaches to alleviate the issue of false positives, as a specific approach may incorrectly identify an interaction [47]. This has produced a protein-protein interaction network with 4918 proteins out of 25831 found in the complete list of reviewed proteins from the UniProt database at http://www.uniprot.org/ and 9707 interactions out of 29430 combined interactions from these protein interaction databases. Protein annotations were retrieved via GOA-UniProtKB [13] using UniProt protein accessions.

For our performance evaluation, we only used proteins annotated with BP terms in the network produced. This is because two proteins that interact physically are more likely to be involved in similar biological processes [40] but there is no guarantee that they share molecular functions [48]. Among 25831 proteins found in the complete list of reviewed proteins in human, 10620 proteins are annotated with GO BP terms. After removing all uncharacterized proteins with respect to the BP ontology from the network, 6417 direct interactions remain if we exclude annotations inferred electronically (IEA) and 7712 direct interactions remain when using all GO evidence codes (http://www.geneontology.org/GO.evidence.shtml). This was used as a positive control set. Lack of complete knowledge about protein interaction sets makes the generation of a negative control set challenging, since the fact that two proteins are not known to interact may simply be because this interaction has not yet been detected [47]. One of the models suggests generating a set of negatives from randomly selecting pairs from all proteins in the dataset under consideration [49, 50]. Thus, negative datasets with equal numbers of protein pairs as in the positive interaction dataset were built by randomly choosing annotated human protein pairs in the proteome. In our context, this is relevant as the probability of randomly selecting a true protein-protein interaction is very low (less than 0.052%).

**Figure 4:**
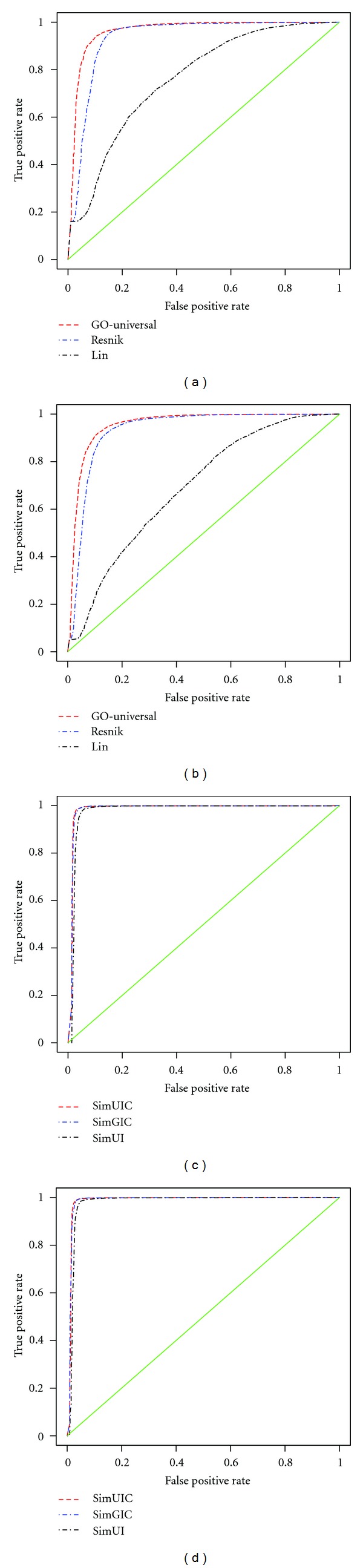
ROC evaluations of functional similarity approaches based on the human PPI dataset derived from different PPI databases.

The classification power of the new metric was tested by receiver operator characteristic (ROC) curve analysis [51] which measures the true positive rate or sensitivity against the false positive rate or 1-specificity. The best match average version of the new metric is compared to the best match average under the Lin measure and that using the Resnik measure which has been shown to perform better than others [52]. Our functional similarity measure inferred using Jaccard index weighted by topological information (SimUIC) is compared to SimGIC and SimUI. The SimUI approach refers to the union-intersection protein similarity measure, which is also implemented in the GOstats package [24]. It is a particular case of simGIC or SimUIC which assumes that all GO terms occur at equal frequency, in which case, only the topology of the GO-DAG is needed. This implies that the SimUI approach assigns equal semantic value or information content to all terms in the GO-DAG. The area under the ROC curve (AUC) is used as a measure of discriminative power, the larger the upper AUC value, the more powerful the measure is, and a realistic classifier must have an AUC larger than 0.5. Results found using the ROCR package under the R programming language [53, 54] are shown in Figures 4(a) and 4(b) for the BMA approach and Figures 4(c) and 4(d) for measures inferred from the Jaccard index (term-based approaches), and their AUCs and precisions are shown in Table 3.

These results indicate that all the approaches perform well. In the context of term-based approaches, the new approach performs as well as the SimGIC approach, which is the best annotation-based measure in this case, in terms of AUC, but it performs slightly better than the SimGIC approach in terms of precision excluding IEA and accuracy. When considering protein functional similarity approaches derived from GO term semantic similarity scores (first three rows of Table 3), the new approach outperforms the best annotation-based approach, namely, BMA under Resnik, particularly in precision, and accuracy. This also shows that the new metric is less sensitive to outliers compared to annotation-based approaches, on top of the fact that it only uses the intrinsic topology (structure) of the GO-DAG without requiring annotation data. Thus, the new metric performs better overall than the existing approaches, specifically providing the best performances in the context of annotation-based approaches, namely, BMA under Resnik and SimGIC. Note that the performance of Resnik and SimGIC approaches is related to the corpus under consideration because of its dependence on the frequencies of GO term occurrences in the corpus. This shallow annotation problem constitutes a serious drawback to these approaches, specifically for organisms with sparse GO annotations [55] and may negatively affect their performances [52]. The use of the whole set of annotations may solve this problem but could, in turn, increase the complexity of these annotation-based approaches as the number of protein annotations increases daily. This would potentially hamper the performance of these approaches in their running time, since reading the annotation file takes time.

Looking at the two main groups of protein functional similarity approaches, term-based approaches perform better than those using GO term semantic similarity scores. This is in part due to the fact that models of protein functional similarity approaches using GO term semantic similarity scores are based on statistical measures of closeness (Avg, Max), which are known to be sensitive to scores that lie at abnormal distances from the majority of scores, or outliers. This means that these measures may produce biases which affect protein functional similarity scores. Furthermore, we investigate if the performance can be improved by leaving out GO annotations with IEA evidence codes. Interestingly, no significant improvement is achieved when leaving out GO annotations with IEA evidence code suggesting that these IEA annotations are in fact of high quality [33, 56]. This also justifies observations made by Guzzi et al. [52] concerning the use of all types of GO evidence codes when assessing a given GO-based semantic similarity approach. Finally, as expected among term-based approaches, SimUIC and SimGIC approaches perform better than the SimUI approach. 

### 3.3. Comparison of the GO-Universal Metric with State-of-the-Art Measures

 We assess the effectiveness of the new metric compared to other topology-based approaches, namely, the Wang and Zhang approaches, the Resnik-related functional similarity measures, and SimGIC. We used a dataset of proteins with known relationships downloaded from the Collaborative Evaluation of Semantic Similarity Measures (CESSMs) online tool [57] at http://xldb.di.fc.ul.pt/tools/cessm/. The set of interacting proteins was extracted from UniProt [58, 59] with GO annotations being obtained from GOA-UniProtKB [13]. CESSM is an online tool for evaluating protein GO-based semantic similarity measures or functional similarity metrics, integrating several functional similarity approaches. The CESSM tool has made the comparison of new semantic measures against previously developed annotation-based metrics possible using Pearson's correlation measures with sequence, Pfam domain and Enzyme Commission (EC) similarity, as well as measuring resolution. Correlation measures how effective the new approach is in capturing sequence, Pfam, and EC similarity. Resolution, which is defined as the relative intensity with which variations in the sequence similarity scale are translated into the semantic similarity scale, provides an indication of how sensitive the approach is to differences in the annotations [36]. This implies that a metric with a higher correlation and resolution performs better, since it captures sequence, Pfam, and EC similarity well and it is likely to be an unbiased metric.

To evaluate the new metric, we ran the CESSM online tool and results are shown in Table 4 for BP and MF. These results indicate that our approach effectively captures sequence, Pfam, and EC similarity in terms of Pearson's correlation, especially for the BP ontology. According to the Pesquita et al. performance classification [36], the SimGIC measure provides the best overall performance among all annotation-based approaches, followed by the Resnik under BMA approach. For the BP ontology, overall our approach outperforms the existing annotation-based approaches, by appearing in the top two measures for all four parameters tested, unlike any of the other measures. It consistently shows one of the highest correlation with sequence, Pfam and EC similarity and also provides one of the two best resolutions, thus achieving overall best performance. For the MF ontology, our approach generally performs well producing good Pearson's correlation compared to the existing annotation-based approaches, and specifically outperforming existing annotation-based approaches in terms of EC and Pfam similarity. It is among the top measures for three out of four parameters, specifically providing high resolution under SimUIC. The new approach consistently outperforms the Wang and Zhang approaches, except for resolution, where the Wang et al. approach performs marginally better for BP. Overall, this shows the improved consistency and relevance of the new metric compared to the existing ones, and our approach has the advantage of being independent of annotation data.

### 3.4. Assessing Functional Similarity between Protein Orthologues Using the GO-Universal Metric

 Orthologous proteins in different species are thought to maintain similar functions. Therefore, we used protein sequence data together with protein GO annotations to determine the extent to which sequence similarities between protein orthologues are translated into similarities between their GO annotations through the GO-universal metric using protein orthologues between human (*Homo sapiens*) and mouse (*Mus musculus* strain C57BL/6) as a case study. Protein orthologue pairs were retrieved from the Ensembl website [60, 61] at http://www.ensembl.org/index.html, GO-association data were downloaded from the GOA site, and the protein sequence files were retrieved from UniProtKB [58, 59, 62].

In order to produce sequence similarity data, an all-against-all BLASTP [63, 64] was performed under the BLOSUM62 amino acid substitution matrix [65]. We obtained BLAST bit scores of these pairwise orthologues in order to compute their sequence similarity scores using the approach suggested in [66]. After removing protein pairs with at least one nonannotated protein, 10691 protein pairs annotated with molecular function terms and 10675 pairs with biological process terms remained. We investigated the power of the GO-universal metric to assess functional similarity between orthologues. We found that 82% of orthologue pairs shared high functional similarity (score ≥ 0.7) in MF annotation and 76% in BP annotation. These results are shown in Table 5, together with proportions achieved by the Resnik approach when using all GO evidence codes, as well as results for both approaches when leaving out IEA and ISS (inferred from sequence or structural similarity) evidence codes. The number of ortholog pairs with GO annotations when IEA and ISS annotations are removed drops to less than 4000 pairs, and the percentage of these pairs sharing high functional similarity drops significantly, particularly for BP. The negative impact of removing IEA annotations has been reported previously [52] and may be due to the fact that IEA and ISS annotations tend to be to higher level GO terms compared to manual mappings.

The high proportion of functionally similar protein orthologues observed in the full dataset was expected, since many of the GO annotations probably arose from homology-based annotation transfer [67, 68]. We were also interested in finding orthologues with very low protein functional similarity scores based on their GO annotations. The new metric was able to detect such cases, which are contrary to the belief in function conservation between orthologues. Some examples are shown in Table 6 together with their GO annotations, GO evidence codes, and sources. There are several possible reasons for this, including protein misannotations, the use of more general GO terms for one and more specific terms for the other protein, or simply the lack of relevant biological knowledge about these proteins. For biological process, in particular, in the examples in Table 6, the differing terms are not conflicting processes, so it may be that the other terms are correct but have just not yet been added, or they may be organism specific. This example provides an illustration of a biological application of the metric and how it can be used to identify possible incorrect or missing annotations.

## 4. Conclusions

 In this work, we have set up a new approach to measure the closeness of terms in the gene ontology (GO), thus translating the difference between the biological contents of terms into numeric values using topological information shared by these terms in the GO-DAG. Like other measures, this enables us to measure functional similarities of proteins on the basis of their GO annotations derived from heterogeneous data sources using semantic similarities of their GO terms. We compare our method to two similar measures and show its advantages. The similarity measure which we defined shows consistent behaviour in that going down the DAG (away from the root) increases specificity, thus providing an effective semantic value for GO terms that reflects functional relationships between GO annotated proteins.

The relevance of this measure is evident when considering the GO hierarchy, as it makes explicit use of the two main relationships between different terms in the DAG, which makes it possible to provide a more precise view of the similarities between terms. This measure yields a simple and reliable semantic similarity between GO terms and functional similarity measure for sets of GO terms or proteins. We have validated this new metric using ROC analysis on human PPI datasets and a selected protein dataset from UniProt with their GO annotations obtained from GOA-UniProt and analysis by the Collaborative Evaluation of Semantic Similarity Measures (CESSM) online tool. Results show that this new GO-semantic value measure that we have introduced constitutes an effective solution to the GO metric problem for the next generation of functional similarity metrics.

As a biological use case, we have applied the GO-universal metric to determine functional similarity between orthologues based on their GO annotations. In most cases functional conservation was shown, but we did identify some orthologues annotated with different functions. This suggests that the new metric can be used to track protein annotation errors or missing annotations. We are currently applying it to assess the closeness of InterPro entries using their mappings to GO. This measure will also be used to design a retrieval tool for genes and gene products based on their GO annotations, providing a new tool for gene clustering and knowledge discovery on the basis of GO annotations. Given a source protein or a set of GO terms, this engine will be able to retrieve functionally related proteins from a specific proteome based on their functional closeness, or identify genes and gene products matched by these functions or very similar functions.

## Supplementary Material

The supplementary material describes the properties of the GO similarity measure, and provides evidence that it is a metric.

## Figures and Tables

**Table 1:** Names and characteristics of GO terms in Figure 3, including topological position characteristics *μ* and information content IC_*T*_ from our approach and IC_*Z*_ and IC_*Zu*_ from the Zhang et al. approach.

GO Id	Level	*μ*	IC_*T*_	IC_*Z*_	IC_*Zu*_
GO:0042770	6	0.0456910e-27	6.525565e+01	10.11006	0.71747
GO:0042772	7	0.1142274e-28	6.664195e+01	12.30729	0.87340
GO:0030330	7	0.1142274e-28	6.664195e+01	11.20867	0.79544
GO:0000077	7	0.0171747e-34	8.235221e+01	10.92099	0.77502
GO:0008630	10	0.0335723e-86	2.014164e+02	12.30729	0.87340
GO:0006978	8	0.0434930e-57	1.343825e+02	12.30729	0.87340
GO:0006977	9	0.0419985e-79	1.850743e+02	12.30729	0.87340
GO:0042771	11	0.1278292e-116	2.691569e+02	12.30729	0.87340
GO:0031571	8	0.1103023e-50	1.173338e+02	12.30729	0.87340
GO:0031572	8	0.0735349e-50	1.177393e+02	12.30729	0.87340
GO:0031573	8	0.4293676e-36	8.373851e+01	12.30729	0.87340
GO:0031574	8	0.2206046e-50	1.166406e+02	12.30729	0.87340

**Table 2:** Semantic similarity values between child-parent pairwise terms in Figure 3 from the Wang et al. and Zhang et al. approaches are compared to our approach. *S*_*W*_ refers to the semantic similarity between two GO terms obtained using the Wang semantic similarity approach from G-SESAME (Gene Semantic Similarity Analysis and Measurements) Tools. *D* values, *S*_*Z*_, *S*_*ZuR*_, and *S*_*ZL*_ refer to the Zhang et al. approach and *S*_GO_ refers to the semantic similarity approach developed here.

Parent GO Id	Child GO Id	*S*_GO_	*S*_*W*_	*S*_*Z*_	*S*_*ZuR*_	*S*_*ZL*_
GO:0042770	GO:0042772	0.97920	0.940	10.11006	0.71747	0.90199
GO:0042770	GO:0030330	0.97920	0.940	10.11006	0.71747	0.94847
GO:0042770	GO:0008630	0.32398	0.704	10.11006	0.71747	0.90199
GO:0042770	GO:0000077	0.79240	0.802	10.11006	0.71747	0.96144
GO:0042772	GO:0006978	0.49591	0.882	12.30729	0.87340	1.00000
GO:0030330	GO:0006978	0.49591	0.889	11.20867	0.79544	0.95328
GO:0030330	GO:0006977	0.36008	0.615	11.20867	0.79544	0.95328
GO:0030330	GO:0042771	0.24760	0.696	11.20867	0.79544	0.95328
GO:0008630	GO:0042771	0.74832	0.931	12.30729	0.87340	1.00000
GO:0000077	GO:0031571	0.70186	0.830	10.92099	0.77502	0.94032
GO:0000077	GO:0031572	0.69945	0.850	10.92099	0.77502	0.94032
GO:0000077	GO:0031573	0.98344	0.948	10.92099	0.77502	0.94032
GO:0000077	GO:0031574	0.70603	0.870	10.92099	0.77502	0.94032
GO:0031571	GO:0006977	0.63398	0.774	12.30729	0.87340	1.00000

**Table 3:** Area under ROC curves (AUCs) and precision for the human PPI dataset. For each group, the top score is in bold.

Approaches	Area under curve (AUC)		Precision		Accuracy	
	Excluding IEA	Including IEA	Excluding IEA	Including IEA	Excluding IEA	Including IEA
GO-universal	**0.962**	**0.954**	**0.841**	**0.772**	**0.885**	**0.816**
Resnik	0.933	0.931	0.724	0.701	0.713	0.739
Lin	0.763	0.691	0.610	0.568	0.481	0.549

SimUIC	**0.983**	**0.986**	**0.930**	0.916	**0.977**	**0.979**
SimGIC	**0.983**	**0.986**	0.922	**0.917**	0.974	0.974
SimUI	0.975	0.978	0.866	0.845	0.926	0.937

**Table 4:** Comparison of performance of our approach with Wang et al., Zhang et al. and annotation-based ones using Pearson's correlation with enzyme Commission (eC), Pfam and sequence similarity, and resolution. Results are obtained from the CESSM online tool. For each ontology, the top two best scores among 12 approaches are in bold.

Ontology	Approaches		Similarity measure correlation			Resolution
			EC	PFAM	Seq Sim	
BP	GO-Universal	(BMA)	**0.44287**	**0.53919**	**0.76797**	**0.90067**
	Wang et al.		0.43266	**0.46692**	0.63356	**0.90966**
	Zhang et al.		0.21944	0.26495	0.20270	0.30148
	Resnik	Avg	0.30218	0.32324	0.40685	0.33673
		Max	0.30756	0.26268	0.30273	0.64522
		BMA	**0.44441**	0.45878	0.73973	0.90041
	Term-based	SimUIC	0.38458	0.43693	0.74410	0.84503
		SimGIC	0.39811	0.45470	**0.77326**	0.83730

MF	GO-Universal	(BMA)	**0.73886**	0.60285	0.55163	0.52905
	Wang et al.		**0.65910**	0.49101	0.37101	0.33109
	Zhang et al.		0.49753	0.41147	0.32235	0.39865
	Resnik	Avg	0.39635	0.44038	0.50143	0.41490
		Max	0.45393	0.18152	0.12458	0.38056
		BMA	0.60271	0.57183	**0.66832**	**0.95771**
	Term-based	SimUIC	0.65826	**0.62510**	0.60512	**0.96928**
		SimGIC	0.62196	**0.63806**	**0.71716**	0.95590

**Table 5:** Proportion in percentage of Human-Mouse orthologue pairs sharing high functional similarity.

	Using all GO evidence codes		Leaving out IEA and ISS	
Approach	BP	MF	BP	MF
GO-Universal	76	82	12	49
Resnik	76	80	13	38

**Table 6:** Some human-mouse protein orthologue pairs without GO-based functional similarity.

	Protein ID	Organism	Annotation information			
			GO ID	GO name	Code	Source
BP	A1Z1Q3	Homo sapiens	GO:0042278	Purine nucleoside metabolic process	IDA	UniProtKB
	Q3UYG8	Mus musculus	GO:0007420	Brain development	IEP	UniProtKB
	Q96EQ8	Homo sapiens	GO:0032480	Negative regulation of type I interferon production	TAS	Reactome
			GO:0045087	Innate immune response	TAS	Reactome
	Q9D9R0	Mus musculus	GO:0016567	Protein ubiquitination	EXP	GOC
	O00451	Homo sapiens	GO:0007169	Transmembrane receptor protein tyrosine kinase signaling pathway	TAS	PINC
			GO:0035860	Glial cell-derived neurotrophic factor receptor signaling pathway	TAS	GOC
	O08842	Mus musculus	GO:0007399	Nervous system development	IMP	MGI
	Q9BS16	Homo sapiens	GO:0000087	M phase of mitotic cell cycle	TAS	Reactome
			GO:0000236	Mitotic prometaphase	TAS	Reactome
			GO:0000278	Mitotic cell cycle	TAS	Reactome
			GO:0006334	Nucleosome assembly	TAS	Reactome
			GO:0034080	Cenh3-containing nucleosome assembly at centromere	TAS	Reactome
	Q9ESN5	Mus musculus	GO:0045944	Positive regulation of transcription from RNA polymerase II promoter	IDA	MGI
	O15347	Homo sapiens	GO:0006310	DNA recombination	ISS	UniProtKB
			GO:0007275	Multicellular organismal development	TAS	PINC
	O54879	Mus musculus	GO:0045578	Negative regulation of B cell differentiation	IDA	MGI
			GO:0045638	Negative regulation of myeloid cell differentiation	IDA	MGI
	Q9NP31	Homo sapiens	GO:0001525	Angiogenesis	IEA	UniProtKB
			GO:0007165	Signal transduction	TAS	PINC
			GO:0007275	Multicellular organismal development	IEA	UniProtKB
			GO:0030154	Cell differentiation	IEA	UniProtKB
	Q9QXK9	Mus musculus	GO:0008283	Cell proliferation	IMP	occurs_in (CL:0000084)
	Q9C035	Homo sapiens	GO:0009615	Response to virus	IEA	UniProtKB
			GO:0044419	Interspecies interaction between organisms	IEA	UniProtKB
			GO:0070206	Protein trimerization	IDA	UniProtKB:Q9C035-1
	P15533	Mus musculus	GO:0006351	Transcription, DNA-dependent	IEA	UniProtKB
			GO:0006355	Regulation of transcription, DNA-dependent	IEA	UniProtKB

MF	Q86XR7	Homo sapiens	GO:0004871	Signal transducer activity	IMP	UniProtKB
	Q8BJQ4	Mus musculus	GO:0005515	Protein binding	IPI	BHF-UCL
	Q99218	Homo sapiens	GO:0030345	Structural constituent of tooth enamel	IDA	BHF-UCL
	P63277	Mus musculus	GO:0005515	Protein binding	IPI	MGI, BHF-UCL
			GO:0008083	Growth factor activity	IMP	BHF-UCL
			GO:0042802	Identical protein binding	IPI	BHF-UCL
			GO:0043498	Cell surface binding	IMP	BHF-UCL
			GO:0046848	Hydroxyapatite binding	IDA	BHF-UCL
	P45379	Homo sapiens	GO:0003779	Actin binding	IDA	UniProtKB
			GO:0005523	Tropomyosin binding	IDA	UniProtKB
			GO:0030172	Troponin C binding	IPI	UniProtKB
			GO:003113	Troponin I binding	IPI	UniProtKB
			GO:0016887	Atpase activity	IDA	UniProtKB:P45379-1-6-7-8
	P50752	Mus musculus	GO:0005200	Structural constituent of cytoskeleton	IDA	occurs_in (CL:0000193)
	Q9H0E3	Homo sapiens	GO:0003713	Transcription coactivator activity	IDA	UniProtKB
			GO:0004402	Histone acetyltransferase activity	IDA	UniProtKB
	Q8BIH0	Mus musculus	GO:0005515	Protein binding	IPI	UniProtKB
	Q5T9L3	Homo sapiens	GO:0004871	Signal transducer activity	ISS	UniProtKB
	Q6DID7	Mus musculus	GO:0005515	Protein binding	IPI	UniProtKB
			GO:0017147	Wnt-protein binding	IDA	UniProtKB
	A8CG34	Homo sapiens	GO:0005515	Protein binding	IPI	UniProtKB
	Q8K3Z9	Mus musculus	GO:0017056	Structural constituent of nuclear pore	IEA	ENSEMBL
	O15446	Homo sapiens	GO:0003899	DNA-directed RNA polymerase activity	IEA	UniProtKB
	Q76KJ5	Mus musculus	GO:0005515	Protein binding	IPI	MGI
