# Supplementary material for: A Topology-Based Metric for Measuring Term Similarity in the Gene Ontology
Source: Adv Bioinformatics. 2012 May 15;2012:975783. doi: 10.1155/2012/975783 (PMC3361142; doi:10.1155/2012/975783)
Supplement: Supplementary file 1 — The supplementary material describes the properties of the GO similarity measure, and provides evidence that it is a metric. [file 975783.f1.pdf]

A Topology-based Metric for Measuring Term Similarity in the Gene Ontology (GO): Supporting Information

Gaston K. Mazandu and Nicola J. Mulder*

Computational Biology Group/ Department of Clinical Laboratory Sciences, Health Sciences
Universisty of Cape Town, South Africa, August 2011.
*Email: nicola.mulder@uct.ac.za

Properties of the GO-universal similarity measure and the metric induced

Given two GO terms x and y, the GO-universal similarity measure $\mathcal{S}_{GO}\left(x,y\right)$ clearly ranges between 0 and 1, i.e., $0 \leq \mathcal{S}_{GO}\left(x,y\right) \leq 1$, and for any GO-terms x, y, and z in the GO-DAG, $\mathcal{S}_{GO}$ satisfies the following property:

$$\mathcal{S}_{GO}\left(x,z\right) + \mathcal{S}_{GO}\left(z,y\right) \leq 1 + \mathcal{S}_{GO}\left(x,y\right) \quad (1)$$

The more general minimum spanning tree structure representing the three GO terms x, y, and z in the GO DAG is given by supplementary figure 1, and other cases are mapped to it. For these three GO terms, the following 6 cases are possible: $IC_T(z) \leq IC_T(y) \leq IC_T(x)$, $IC_T(z) \leq IC_T(x) \leq IC_T(y)$, $IC_T(y) \leq IC_T(z) \leq IC_T(x)$, $IC_T(y) \leq IC_T(x) \leq IC_T(z)$, $IC_T(x) \leq IC_T(z) \leq IC_T(y)$ or $IC_T(x) \leq IC_T(y) \leq IC_T(z)$. Let's consider the first case, if $IC_T(z) \leq IC_T(y) \leq IC_T(x)$ then $\max\{IC_T\left(x\right), IC_T\left(y\right)\} = IC_T(x)$, $\max\{IC_T\left(x\right), IC_T\left(z\right)\} = IC_T(x)$, and $\max\{IC_T\left(y\right), IC_T\left(z\right)\} = IC_T(y)$. From figure 1, we have $\frac{IC_T(x,z)}{IC_T(x)} \leq \frac{IC_T(x,y)}{IC_T(x)}$ and as $\frac{IC_T(y,z)}{IC_T(y)} \leq 1$, it follows that

$$\frac{IC_T(x,z)}{IC_T(x)} + \frac{IC_T(y,z)}{IC_T(y)} \leq 1 + \frac{IC_T(x,y)}{IC_T(x)}$$

Finally putting everything together, we have:

$$\frac{IC_T(x,z)}{\max\{IC_T\left(x\right), IC_T\left(z\right)\}} + \frac{IC_T(y,z)}{\max\{IC_T\left(y\right), IC_T\left(z\right)\}} \leq 1 + \frac{IC_T(x,y)}{\max\{IC_T\left(x\right), IC_T\left(y\right)\}}$$

Meaning that

$$\mathcal{S}_{GO}\left(x,z\right) + \mathcal{S}_{GO}\left(z,y\right) \leq 1 + \mathcal{S}_{GO}\left(x,y\right)$$

The same reasoning can be applied to the other cases.

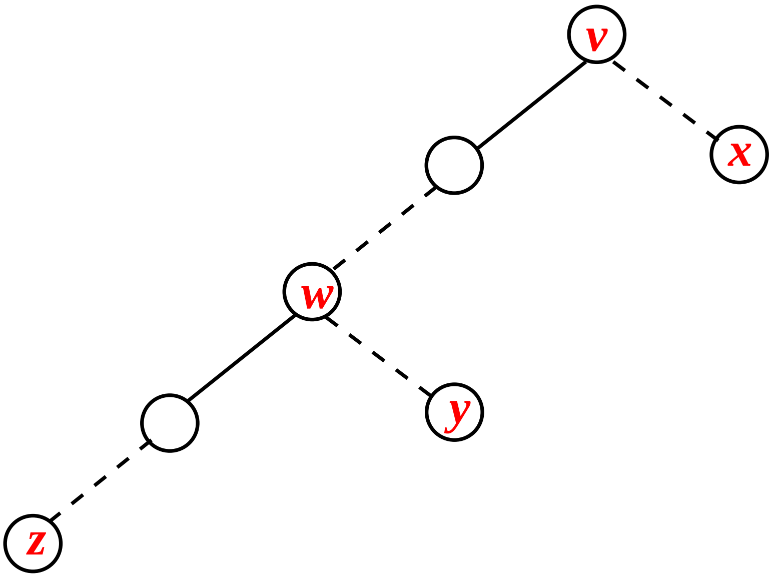

Figure 1: **General structure of minimum spanning tree for 3 GO terms x, y and z in the GO DAG**. This provides a general representation of 3 GO terms in the GO DAG with a minimum number of edges in which - - - and —— are links inferred from the structure. - - - means that the branches can go down as low as they can and —— shows the possible direct links.

The quantity $d_{GO}(x, y) = 1 - \mathcal{S}_{GO}(x, y)$ satisfying $0 \leq d_{GO}(x, y) \leq 1$ defines a metric or distance on $\mathcal{N}_{GO}$. The following properties are satisfied:
(i) Positive definiteness: as $0 \leq \mathcal{S}_{GO}(x, y) \leq 1$, we have $1 - \mathcal{S}_{GO}(x, y) \geq 0$ meaning that $d_{GO}(x, y) \geq 0$.
(ii) Symmetry axiom: as $\mathcal{S}_{GO}(x, y) = \mathcal{S}_{GO}(y, x)$, we have $1 - \mathcal{S}_{GO}(x, y) = 1 - \mathcal{S}_{GO}(y, x)$, which means that $d_{GO}(x, y) = d_{GO}(y, x)$.
(iii) Identity axiom: $d_{GO}(x, y) = 0$ implies $1 - \mathcal{S}_{GO}(x, y) = 0$, meaning that $S_{GO}(x, y) = 1$, which implies that $x \overset{\text{GO}}{=} y$.
(iv) Finally, sub-additivity or triangle inequality: from the relation (1), we know that $0 \leq \mathcal{S}_{GO}(x, z) + \mathcal{S}_{GO}(z, y) \leq 1 + \mathcal{S}_{GO}(x, y)$. It follows that $-[1 + \mathcal{S}_{GO}(x, y)] \leq -[\mathcal{S}_{GO}(x, z) + \mathcal{S}_{GO}(z, y)]$. Adding 2 on both sides, we have $1 - \mathcal{S}_{GO}(x, y) \leq [1 - \mathcal{S}_{GO}(x, z)] + [1 - \mathcal{S}_{GO}(z, y)]$, which means that $d_{GO}(x, y) \leq d_{GO}(x, z) + d_{GO}(z, y)$.
It follows that d_{GO} is a metric and this metric is referred to as the GO-universal metric.
